# Chemotherapy Shifts the Balance in Favor of CD8+ TNFR2+ TILs in Triple-Negative Breast Tumors

**DOI:** 10.3390/cells10061429

**Published:** 2021-06-08

**Authors:** Tamir Baram, Nofar Erlichman, Maya Dadiani, Nora Balint-Lahat, Anya Pavlovski, Tsipi Meshel, Dana Morzaev-Sulzbach, Einav Nili Gal-Yam, Iris Barshack, Adit Ben-Baruch

**Affiliations:** 1 George S. Wise Faculty of Life Sciences, The Shmunis School of Biomedicine and Cancer Research, Tel Aviv University, Tel Aviv 69978-01, Israel; tamiros21@gmail.com (T.B.); nofarerlichman@gmail.com (N.E.); tsipi.meshel@gmail.com (T.M.); 2 Sheba Medical Center, Breast Oncology Institute, Ramat Gan 5211401, Israel; Maya.Dadiani@sheba.health.gov.il (M.D.); Dana.Morzaev@sheba.health.gov.il (D.M.-S.); Einav.NiliGal-Yam@sheba.health.gov.il (E.N.G.-Y.); 3 Sheba Medical Center, Pathology Institute, Ramat Gan 5211401, Israel; Nora.BalintLahat@sheba.health.gov.il (N.B.-L.); anya.pavlovsky@gmail.com (A.P.); Iris.Barshack@sheba.health.gov.il (I.B.); 4 Sackler School of Medicine, Tel Aviv University, Tel Aviv 69978-01, Israel

**Keywords:** CD4+ lymphocytes, CD8+ lymphocytes, forkhead box P3 (FOXP3), programmed cell death protein 1 (PD-1), splenocytes, triple-negative breast cancer (TNBC), tumor-infiltrating lymphocytes (TILs), tumor necrosis factor α (TNFα), tumor necrosis factor receptor 2 (TNFR2)

## Abstract

Triple-negative breast cancer (TNBC) is primarily treated via chemotherapy; in parallel, efforts are made to introduce immunotherapies into TNBC treatment. CD4+ TNFR2+ lymphocytes were reported as Tregs that contribute to tumor progression. However, our published study indicated that TNFR2+ tumor-infiltrating lymphocytes (TNFR2+ TILs) were associated with improved survival in TNBC patient tumors. Based on our analyses of the contents of CD4+ and CD8+ TILs in TNBC patient tumors, in the current study, we determined the impact of chemotherapy on CD4+ and CD8+ TIL subsets in TNBC mouse tumors. We found that chemotherapy led to (1) a reduction in CD4+ TNFR2+ FOXP3+ TILs, indicating that chemotherapy decreased the content of CD4+ TNFR2+ Tregs, and (2) an elevation in CD8+ TNFR2+ and CD8+ TNFR2+ PD-1+ TILs; high levels of these two subsets were significantly associated with reduced tumor growth. In spleens of tumor-bearing mice, chemotherapy down-regulated CD4+ TNFR2+ FOXP3+ cells but the subset of CD8+ TNFR2+ PD-1+ was not present prior to chemotherapy and was not increased by the treatment. Thus, our data suggest that chemotherapy promotes the proportion of protective CD8+ TNFR2+ TILs and that, unlike other cancer types, therapeutic strategies directed against TNFR2 may be detrimental in TNBC.

## 1. Introduction

Triple-negative breast tumors (TNBCs) lack the expression of receptors that can serve as therapeutic targets, thus patients are treated primarily using chemotherapy. Initially, response rates are relatively high, but TNBC tumors are likely to recur and demonstrate a very aggressive phenotype [1,2,3]. Inspired by the relatively high success rate of immunotherapies in malignancies, such as melanoma, recent clinical studies introduced immune checkpoint blockades (ICBs), such as antibodies to PD-1 and PD-L1 [4,5,6] to TNBC treatment [5,6,7,8,9,10]. This approach was driven by the hypothesis that out of the different subtypes of breast cancer, TNBC tumors may be the most suitable for treatment using ICBs because they demonstrate a relatively high mutation rate, which may lead to the expression of neo-antigens and thus to activation of lymphocytes against tumor cells [11,12,13]. In addition, TNBC tumors are characterized by a high presence of tumor-infiltrating lymphocytes (TILs) that are significantly associated with a good prognosis [14,15,16,17]. However, clinical studies that have used ICBs in TNBC patients demonstrated successive tumor growth and relatively low response rates [5,6,7,8,9,10]. The results of these clinical trials call for the improved identification of the immune contexture in TNBC tumors and of the relevance of different TIL subsets to anti-tumor immune activities at the tumor site.

In the context of regular immune activities, lymphocytes that express TNFR2, a receptor for tumor necrosis factor α (TNFα), were demonstrated as regulators of immune activities. Although CD4+ T effector cells were found to express TNFR2 (usually in relatively low levels), most reports identified CD4+ TNFR2+ lymphocytes as being highly potent immuno-suppressive FOXP3-expressing T regulatory cells (Tregs) in humans and mice [18,19,20,21,22,23,24]. It was demonstrated that TNFR2+ Tregs had pronounced abilities to prevent excessive inflammatory processes and balance immune functions [25,26,27,28,29,30].

In parallel, it was reported that TNFR2+ Tregs were abnormally located in human and mouse tumors and that their activities inhibited immune surveillance, leading to elevated tumor aggressiveness [31,32,33,34,35,36,37]. In their study of ovarian carcinoma, Dr. Faustman and her colleagues demonstrated that antagonistic antibodies to TNFR2 limited the expansion of TNFR2+ Tregs and inhibited their functions, with preferential activity toward Tregs of cancer patients than of control subjects [38]. Together, the different studies set the basis for the hypothesis that modalities directed to TNFR2 will reduce the presence and activities of TNFR2+ Tregs in tumors, leading to improved anti-tumor activities that may be exerted by lymphocytes, such as cytotoxic T cells (CTLs) [39,40,41,42,43].

With this information in mind, in our published study, we determined the subset of TNFR2+ TILs in TNBC patient tumors [44]. Unlike studies of TNFR2+ TILs in other tumor types, our research indicated that high proportions of TNFR2+ TILs in TNBC patient biopsies were significantly associated with improved survival [44]. Here, it is important to indicate that in our study, we determined TNFR2+ TILs in patient biopsies taken prior to chemotherapy (adjuvant-treated patients); however, because survival rates are routinely determined after chemotherapy, our results may have reflected the fact that TNFR2+ TILs, which were present in the tumors prior to chemotherapy, were affected by the treatment in a manner that increased anti-tumor activities and contributed to improved survival rates in the patients.

This possibility is supported by reports indicating that chemotherapy can reduce/ablate Tregs in tumors [45,46,47,48,49]. Thus, in the current follow-up study, our working hypothesis was that chemotherapy down-regulated the presence/activities of TNFR2+ Tregs that were present in the tumors prior to chemotherapy, leading to the improved survival of TNBC patients. In parallel, we considered the possibility that another subset of TNFR2+ TILs, which may lead to elevated levels of anti-tumor activities, was selectively preferred during chemotherapy, contributing its share to better outcomes in the patients.

Thus, in the current study, we asked what the impact of chemotherapy on CD4+ TNFR2+ TILs was by comparing chemotherapy- and vehicle-treated mice in a syngraft model of 4T1 TNBC cells. Moreover, in view of the few reports on CD8+ TNFR2+ lymphocytes that were identified in malignant and non-malignant conditions (however, not in TNBC) [50,51,52,53], we also analyzed the effect of chemotherapy on CD8+ TNFR2+ TILs. In the course of the study, TNFR2+ TIL subsets were analyzed for the expression of additional markers: FOXP3 (in the case of CD4+ TNFR2+ TILs) and PD-1 (in the case of CD4+ TNFR2+ and CD8+ TNFR2+ TILs). The relevance of PD-1 analysis was guaranteed by using 4T1 cells that expressed PD-L1, in line with many reports describing the expression of PD-L1 in TNBC patient tumors [8,54,55]. Furthermore, to determine whether the phenotype of TILs reflects general events that take place in secondary lymphoid organs, we analyzed the contexture of different TNFR2+ lymphocyte subsets in the spleen in parallel to TILs, in chemotherapy-treated and vehicle-treated mice.

The findings presented in this article demonstrate that chemotherapy induced several alterations in TNFR2+ TIL subsets. Primarily, a significant reduction was observed after chemotherapy in CD4+ TNFR2+ FOXP3+ TILs, reflecting a decrease in the proportions of CD4+ TNFR2+ Tregs. In parallel, elevation in CD8+ TNFR2+ and more so of CD8+ TNFR2+ PD-1+ TILs was noted following chemotherapy, and increased proportions of these two TIL subsets were significantly correlated with a smaller volume of tumors denoted in the chemotherapy-treated group. Because PD-1 reflects a dynamic status in T cell activation—first being up-regulated in activated T cells and then leading to immune suppression by binding to PD-L1 [56,57,58,59]—it is possible that CD8+ TNFR2+ PD-1+ TILs are activated/effector CD8+ cytotoxic T cells; this possibility is supported by recent studies that have identified CD8+ PD-1+ cells as immune-reactive TILs in TNBC and in additional tumor systems [54,60,61], as will be discussed further below.

Thus, the findings of this research identified two new TIL subsets in TNBC tumors consisting of CD8+ TNFR2+ and CD8+ TNFR2+ PD-1+ TILs. They also provide novel findings on the impact of chemotherapy on TNFR2+ TILs subsets, suggesting that beneficial CD8+ TNFR2+/CD8+ TNFR2+ PD-1+ TILs that were present in TNBC tumors before chemotherapy were enriched by chemotherapy at the expense of CD4+ TNFR2+ subsets, such as Tregs; these events may lead to elevated immune surveillance and improved disease outcome in TNBC patients.

## 2. Materials and Methods

### 2.1. Patient Cohort

The study included a cohort of 41 adjuvant-treated TNBC patients. Board-certified pathologists confirmed the TNBC statuses according to ASCO/CAP guidelines. The study was performed in line with the ethical standards and current laws of the country in which it was performed (Israel) and the 1964 Helsinki declaration and its later amendments or comparable ethical standards. The study was approved by the Institutional Review Board of Sheba Medical Center (approval no. 8736-11-SMC) and by the Institutional Ethics Committee of Tel Aviv University. All samples were anonymized, as defined in the study protocol. The clinicopathological characteristics of the patients included in the study are provided in Table 1. In general, patient treatment was initiated up to 6 weeks after surgery, and included 4 treatments of doxorubicin + cyclophosphamide, followed by 4 treatments of paclitaxel; treatments were given every two weeks (total treatment duration: 16 weeks).

### 2.2. Immunohistochemistry

The expression of CD4 and CD8 was determined in formalin-fixed paraffin-embedded tumor sections (4 µm) of TNBC patients using antibodies against human CD4 (#ACI3148A) and human CD8 (#CRM311A) (both from Biocare Medical, Pacheco, CA, USA), which are widely used in the clinic. Tonsils were used as positive controls for staining. CC1 antigen retrieval solution (Ventana, Oro Valley, AZ, USA) was used for heat-induced antigen retrieval in alkaline conditions. Staining patterns were detected using a DAB detection system (Ventana).

The determination of staining patterns was performed using a certified breast pathologist of the Sheba Medical Center in a blind manner. H&E staining was used to assess the tumor histology and identify leukocytes. Stained CD4+ TILs and CD8+ TILs were envisioned in high power field (HPF) view (400×). Data were presented as percentages of CD4+ TILs and CD8+ TILs out of total leukocytes in each specimen. Statistical analyses were performed using two-tailed unpaired Student’s *t*-tests. Values of *p* ≤ 0.05 were considered statistically significant.

### 2.3. PD-L1 Expressing 4T1 Cells

4T1 murine TNBC cells were purchased from ATCC and were grown in a DMEM medium supplemented with 10% fetal bovine serum (FBS) and 1% penicillin-streptomycin solution (all from Biological Industries, Beit Ha’emek, Israel). Following retroviral infection of HEK-293T cells, 4T1 cells were transfected to express murine PD-L1 (mPD-L1). Selection was performed using 2 µg/mL puromycin (#P-1033; AG Scientific, San Diego, CA, USA).

Membrane expression levels of mPD-L1 were determined by Rat IgG2a against mPD-L1 (#14-5982-82, Thermo Fischer Scientific, Waltham, MA, USA), followed by FITC-conjugated goat IgG anti-Rat (#112-095-003, Jackson Immunoresearch, West Grove, PA, USA). Baseline staining was determined by non-relevant isotype-matched antibodies. Flow cytometry analyses were performed by using an S1000EXi flow cytometer (Stratedigm; San Jose, CA, USA) and the Flow-Jo software (version 10; BD Biosciences, San Jose, CA, USA).

### 2.4. Tumor Establishment and Chemotherapy Administration

4T1 cells were orthotopically inoculated to the mammary fat pads of 8–10-weeks-old female BALB/c mice (#BALB/cOlaHsd, Envigo RMS, Jerusalem, Israel). Four days after tumor cell inoculation, chemotherapy was diluted to working concentration in saline and was administrated intraperitoneally to the animals, twice a week, with 3–4-day intervals between administrations. The chemotherapy treatment included Doxorubicin (4 mg/kg; #44583-1MG, Merck KGaA, Darmstadt, Germany) and Paclitaxel (5mg/kg; #T7191-5MG, Merck). Control mice were injected with the vehicle of the chemotherapies (DMSO), which was used in a similar dilution. In line with the regulations of the Ethics Committee, the experiments were terminated on day 19. Procedures involving experimental animals were approved by the Tel Aviv University Ethics Committee (approval no. 04-19-011) and were performed in compliance with local animal welfare laws, guidelines, and policies. Statistical analyses of tumor volumes were performed using two-tailed unpaired Student’s *t*-tests. Values of *p* ≤ 0.05 were considered statistically significant.

### 2.5. Preparation of Dissociated Cells from Tumors and Spleens

On day 19 following the tumor cell inoculation, mice were sacrificed and tumors and spleens were harvested. Volumes of excised tumors were measured using caliper and calculated by the formula V = L × W^2^ × 0.5. Tumors were mechanically dissociated in C tubes (#130-093-237, Miltenyi Biotech, Bergisch Gladbach, Germany) using a GentleMax dissociator (#130-093-235, Miltenyi Biotec). Enzymatic digestion of tumors was performed using Tumor Dissociation Kit (#130-096-730, Miltenyi Biotec) according to the manufacturer’s protocol. Erythrocytes were removed using hypotonic shock. Excised spleens were dissociated manually, and erythrocytes were removed using hypotonic shock. Dissociated tumor and spleen cells were strained in a Falcon 40 µm cell strainer (#352340, Corning, NY, USA) and suspended in a staining buffer containing PBS supplemented with 4% FBS, 4 mM EDTA, 25 mM HEPES buffer, and 0.1% sodium azide.

### 2.6. Analysis of Lymphocyte Subsets

Prior to staining using antibodies, cells that were isolated from tumors (including tumor cells and TILs) and spleens were incubated with FC blocker TruStain FcX (#101319, BioLegend, San Diego, CA, USA) for 10 min at 4 °C. The staining procedure began by incubating the cells with fluorophore-conjugated antibodies to surface markers for 45 min at 4 °C. The following antibodies were used (all from BioLegend): CD45-APC-Cy7 (#103115), CD3-APC (#100235), CD4-BV510 (#100553), CD8-PE-Cy7 (#100721) TNFR2-PE (#113405), and PD-1-BV605 (#135219); cells that underwent the same procedure but were not stained by antibodies were used in parallel.

Following a wash with PBS containing 0.1% sodium azide, cells stained for surface markers (and the unstained cells in parallel) were subjected to intracellular staining of FOXP3. To this end, the cells were incubated in True-Nuclear Transcription Factor Buffer Set (#424401, BioLegend), fixed for 45 min with a buffer provided in the set, washed twice with permeabilization buffer (provided in the set), and incubated for 45 min in permeabilization buffer containing antibodies to FOXP3 (FOXP3-BV421; #126419, BioLegend) or isotype-matched non-relevant control antibodies (IgG2b-BV421; #400639, BioLegend). Following this step of intracellular staining, the cells were washed with permeabilization and staining buffers (PBS supplemented with 4% FBS, 4 mM EDTA, 25 mM HEPES buffer), then suspended in a staining buffer for cell acquisition in flow cytometry.

Prior to cell acquisition in flow cytometry, compensation for multi-color staining was performed using UltraComp eBeads (#01-2222-42, Thermo Fischer Scientific). During cell acquisition, small-sized debris and dead cell particles were excluded via appropriate tuning of FSC/SSC channels. Because the cells analyzed in this study were fixed, gating strategies were determined using several preliminary analyses of cells from tumors and spleens of tumor-bearing mice and cells derived from the spleens of normal non-tumor-bearing mice in parallel. Based on these preliminary analyses, an FSC/SSC gate was set in order to exclude cellular aggregates and as many tumor cells as possible from the analysis (Figure S1, left column). Then, FSC-LinH/FSC-LinA gating was performed in order to preferentially analyze single cells (Figure S1, second column from the left). Figures S1 and S2 provide additional information on the gating strategies used to determine the marker-positive cells. Flow cytometry was performed using an S1000EXi instrument (Stratedigm), and analyses were performed using the Flow-Jo software (version 10; BD Biosciences).

Statistical analyses of lymphocyte subsets were performed using two-tailed unpaired Student’s *t*-tests. In the correlation analysis, Pearson’s correlation coefficient was determined. The significance of the correlation coefficient was calculated using T.DIST. In all analyses, values of *p* ≤ 0.05 were considered statistically significant.

## 3. Results

### 3.1. TNBC Patient Tumors Contained CD4+ TILs and Were Particularly Enriched with CD8+ TILs

The primary aim of the current study was to identify the impact of chemotherapy on TNFR2+ TILs in TNBC-bearing mice. In view of many observations identifying TNFR2+ TILs as CD4+ Tregs, our study addressed CD4+ TILs; in parallel, the study was extended to CD8+ TILs in view of few reports indicating that CD8+ cells can also express TNFR2.

**Figure 1:**
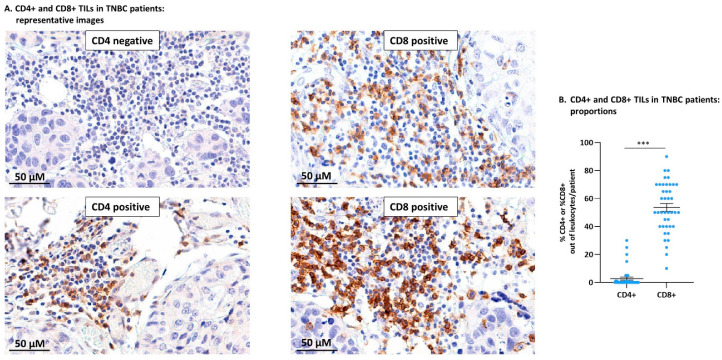
In TNBC patient tumors, CD8+ TILs dominated the T cell landscape over CD4+ TILs. Biopsy sections of TNBC patients were analyzed for the presence of CD4+ and CD8+ TILs using immunohistochemistry. (**A**) Images of representative sections stained for CD4 (two different patients: one negative for CD4+ TILs, the second positive for CD4+ TILs) and CD8 (two different patients: both positive for CD8+ TILs). Bar: 50 µm. (**B**) Semi-quantitative analysis of %CD4+ TILs and %CD8+ TILs out of the leukocyte infiltrate in TNBC patients (n = 41). Data are presented as average ± SE of percentages of the specific subset in all patients. The value of each patient is represented as a dot. *** *p* < 0.001 (two-tailed unpaired Student’s *t*-test).

The analysis of CD4+ TILs and CD8+ TILs in TNBC patient tumors demonstrated the relevance of studying both CD4+ and CD8+ subsets in the mouse TNBC model. The study of TNBC patient tumors (n = 41; clinicopathological characteristics are provided in Table 1) indicated that CD4+, as well as CD8+ TILs, were generally localized in specific tumor areas (Figure 1A). The CD4+ TIL subset was detected only in some of the patients (14/41; Figure 1B) and accounted for 1–30% of the leukocytes in the tumors (Figure 1A demonstrates a CD4-negative tumor biopsy and a CD4-positive biopsy). In parallel, CD8+ TILs were present in all patient biopsies (41/41; Figure 1B) and accounted for 10–80% of the leukocyte population in the different patients (Figure 1A demonstrates CD8-positive tumor biopsies of two different patients).

These findings indicate that CD8+ TILs dominated TNBC patient tumors over CD4+ TILs, thus the CD8+ TIL subset was of major relevance to the impact of the immune contexture on disease progression. These findings were followed by analyses of CD8+ TILs in parallel to CD4+ TILs in murine 4T1 tumors, as described below.

### 3.2. General Characteristics of T Cell Subsets in TILs and Splenocytes of TNBC-Bearing Mice

To determine the impact of chemotherapy on different CD4+ and CD8+ lymphocyte subsets in TNBC tumors, a syngraft system of 4T1 TNBC cells was established. To also identify systemic immune-related events that were affected by chemotherapy, we analyzed the same CD4+ and CD8+ lymphocyte subsets in the spleens of tumor-bearing mice. The analyses were based on the flow cytometry gating strategies demonstrated in Figures S1 and S2 (see also “Section 2”).

**Figure 2:**
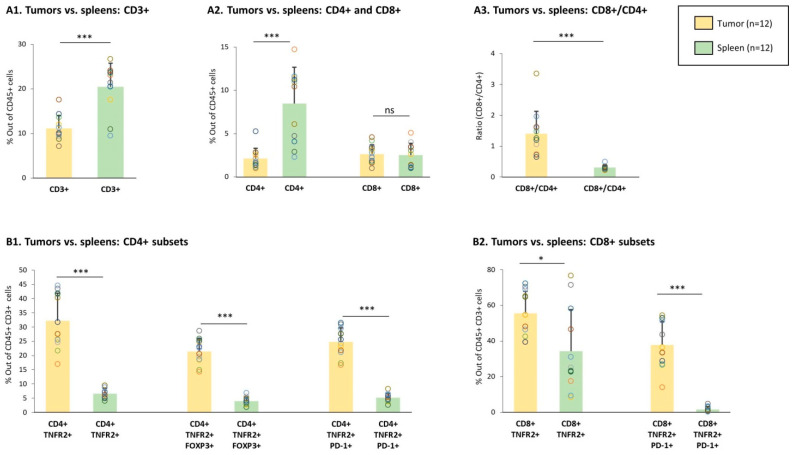
Proportions of lymphocyte sub-populations in TILs and spleens of vehicle-treated tumor-bearing mice. Murine 4T1 TNBC cells were syngrafted to the mammary fat pads of female BALB/c mice. The Figure demonstrates data of TILs and splenocytes from vehicle-treated tumor-bearing mice; n = 12 in total, in 3 biological repeats. (**A**) Proportions of CD3+, CD4+, and CD8+ populations, gated out of CD45+ cells. (**A1**) Percentage of CD3+ cells. (**A2**) Percentages of CD4+ and CD8+ cells. (**A3**) Ratio of CD8+ cells over CD4+ cells, calculated on the basis of the percentages of the two subsets in each mouse. (**B**) Proportions of CD4+ and CD8+ subsets. (**B1**) CD4+ TNFR2+, CD4+ TNFR2+ FOXP3+, and CD4+ TNFR2+ PD-1+ cells, gated out of CD45+ CD3+ cells. (**B2**) CD8+ TNFR2+ and CD8+ TNFR2+ PD-1+ subsets, gated out of CD45+ CD3+ cells. Gating procedures are described in Figures S1 and S2 and in “Section 2”. Data are presented as average ± SD of percentages of the specific subset, in all mice belonging to each group. The data of each mouse are presented as a dot. *** *p* < 0.001, * *p* < 0.05 (two-tailed unpaired Student’s *t*-test).

First, we determined the baseline status of different lymphocyte subsets in the tumors and spleens of vehicle-treated, tumor-bearing mice (not given chemotherapy). The findings of Figure 2A1,A2 indicate that while CD3+ and CD4+ T cells were less abundant in tumors than in spleens, the levels of CD8+ T cells were similar at both sites. Analysis of the ratio between CD8+ T cells and CD4+ T cells indicated that the CD8+/CD4+ ratio was significantly higher in tumors than in spleens (Figure 2A3); the high ratio of CD8+ T cells over CD4+ T cells in the tumors was in line with the dominance of CD8+ TILs over CD4+ TILs in TNBC patients tumors, as demonstrated in Figure 1.

TNFR2+ lymphocytes were then analyzed in tumors and spleens of vehicle-treated TNBC-bearing mice. In the CD4+ arm, we studied: (1) CD4+ TNFR2+ T cells, which accounted for the overall population of CD4+ T cells that expressed TNFR2, (2) CD4+ TNFR2+ FOXP3+ T cells, and (3) CD4+ TNFR2+ PD-1+ T cells; to increase the relevance of this latter analysis, we used PD-L1-expressing 4T1 cells in the study (Figure S3). In parallel, in the CD8+ arm, we studied: (1) the subset of CD8+ TNFR2+ T cells, namely, the overall population of CD8+ T cells that expresses TNFR2 and (2) TNFR2+ CD8+ PD-1+ T cells.

Analyses of these five TNFR2+ lymphocyte subsets in the tumors and spleens of vehicle-treated tumor-bearing mice revealed a significantly higher percentage of all of them—of the CD4+ arm, as well as the CD8+ arm—in TILs compared to splenocytes (Figure 2B1,B2). Of note is the fact that the CD8+ TNFR2+ PD-1+ subset was not present at all in the spleens, whereas a considerable amount of this sub-population was noted in the tumors. Overall, these findings suggest that TNFR2+ lymphocyte subsets were intimately linked to the tumor microenvironment and may participate in the regulation of tumor progression.

### 3.3. Chemotherapy Led to Down-Regulation of CD4+ TNFR2+ TIL Subsets and Up-Regulation of CD8+ TNFR2+ TIL Subsets in Tumors of TNBC-Bearing Mice

**Figure 3:**
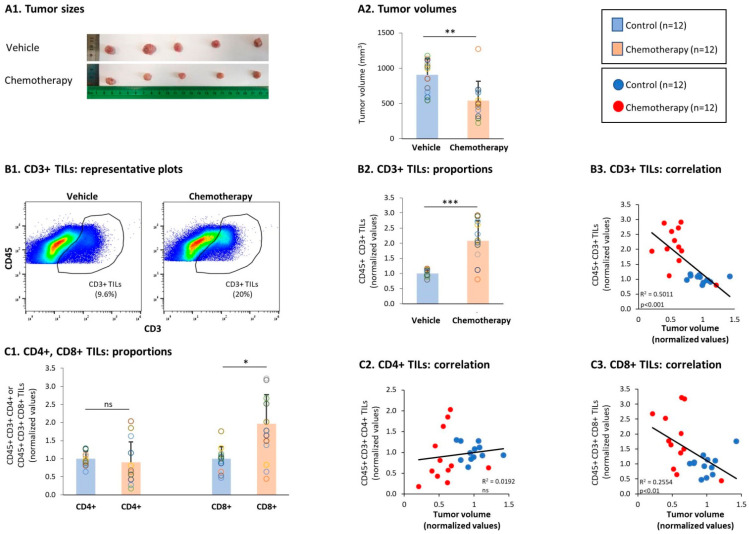
Chemotherapy reduced TNBC tumor growth and elevated the levels of CD3+ and CD8+ TILs. Murine 4T1 TNBC cells were syngrafted to the mammary fat pads of female BALB/c mice; one group of mice received chemotherapy (doxorubicin + paclitaxel) and the other group received the vehicle of the drugs; n = 12 in each group, in total in 3 biological repeats. Chemotherapies were given as described in the Materials and Methods section until the termination of experiments on day 19. (**A**) Tumor sizes. At the end of the experiments, tumor volumes were determined and the tumors were photographed. (**A1**) Tumors excised at the end of the third experiment, demonstrating the tumor sizes in the two groups, n = 5 mice in each group. (**A2**) Average ± SD of tumor volumes in each group. (**B**) CD3+ TILs, gated out of CD45+ cells. (**B1**) Representative flow cytometry analyses of CD3+ TILs. (**B2**) Proportions of CD3+ TILs. (**B3**) CD3+ TILs correlated with tumor volumes. (**C**) CD4+ TILs and CD8+ TILs, gated out of CD45+ CD3+ cells. (**C1**) Proportions of CD4+ TILs and CD8+ TILs. (**C2**,**C3**) CD4+ TILs and CD8+ TILs, respectively, correlated with tumor volumes. Gating procedures are described in Figures S1 and S2 and in “Section 2”. In the proportion and correlation analyses, the average value of the vehicle-treated group in each biological repeat was given the value of 1 and the values of all mice analyzed in that repeat were normalized accordingly. The value of each mouse is presented as a dot. *** *p* < 0.001, ** *p* < 0.01, * *p* < 0.05, ns: not significant (two-tailed unpaired Student’s *t*-test; correlation analyses: Pearson; T.DIST).

To adhere to the chemotherapy regimens given to TNBC patients [48,49], mice were treated using doxorubicin + paclitaxel. Following chemotherapy, a significant reduction in tumor volumes was noted, validating the effectiveness and relevance of the treatment (Figure 3A). Alongside the reduced tumor growth, a significant elevation in the proportion of CD3+ TILs was noted in tumors of chemotherapy-treated mice compared to vehicle-treated mice (Figure 3(B1,B2)). The possible connection of CD3+ TILs with improved survival was revealed via a significant correlation between high proportions of CD3+ TILs and smaller tumor volumes in chemotherapy-treated mice (Figure 3(B3)); moreover, clear dissociation was noticed in this analysis between the group of chemotherapy-treated mice and the group of vehicle-treated mice (Figure 3(B3)).

Analyses of the T cell subsets demonstrated that most of the general CD4+ TIL population was not altered by chemotherapy (Figure 3(C1)); however, the presence of the general CD8+ TIL population was significantly increased by the chemotherapy (Figure 3(C1)). Furthermore, high proportions of CD8+ T cells in the tumors following chemotherapy—but not of CD4+ TILs—were significantly associated with lower tumor sizes (Figure 3(C2,C3)), indicating that activated CD8+ CTLs played a role in reducing the tumor growth.

**Figure 4:**
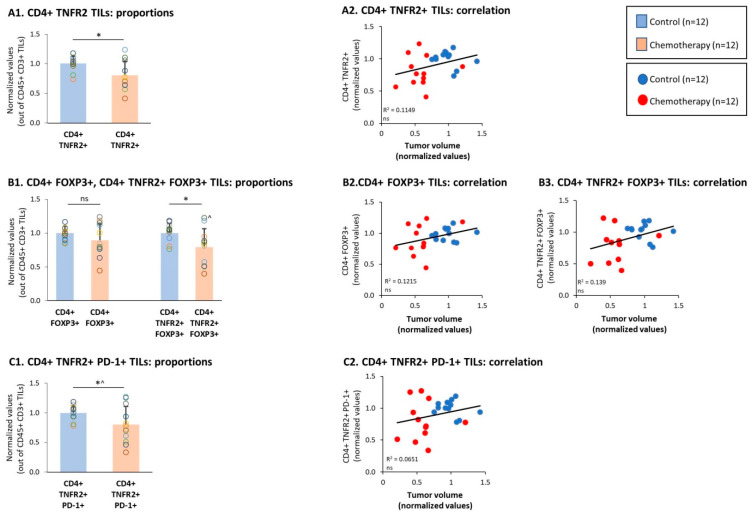
Chemotherapy reduced the proportions of CD4+ TNFR2+ FOXP3+ and CD4+ TNFR2+ PD-1+ TILs. Murine 4T1 TNBC cells were syngrafted to BALB/c mice in the presence of chemotherapy or a vehicle control; procedures and analyses were performed as described in Figure 3. (**A**) CD4+ TNFR2+ TILs, gated out of CD45+ CD3+ cells. (**A1**) Proportions of CD4+ TNFR2+ TILs. (**A2**) CD4+ TNFR2+ TILs, correlated with tumor volumes. (**B**) CD4+ FOXP3+ and CD4+ TNFR2+ FOXP3+ TILs, gated out of the CD45+ CD3+ cells. (**B1**) Proportions of CD4+ FOXP3+ and CD4+ TNFR2+ FOXP3+ TILs. (**B2**, **B3**) CD4+ FOXP3+ and CD4+ TNFR2+ FOXP3+ TILs, respectively, correlated with tumor volumes. In B1: ^ n = 11. (**C**) CD4+ TNFR2+ PD-1+ TILs, gated out of CD45+ CD3+ cells. (**C1**) Proportions of CD4+ TNFR2+ PD-1+ TILs. (**C2**) CD4+ TNFR2+ PD-1+ TILs, correlated with tumor volumes. In the proportion and correlation analyses, the average value of the vehicle-treated group in each biological repeat was given the value of 1 and the values of all mice analyzed in that repeat were normalized accordingly. The value of each mouse is presented as a dot. * *p* < 0.05 (in C1: ^ *p* = 0.0505), ns: not significant (two-tailed unpaired Student’s *t*-test; correlation analyses: Pearson; T.DIST).

Then, analyses of the CD4+ TNFR2+ TILs demonstrated that this subset was significantly reduced by chemotherapy (Figure 4(A1)). Moreover, high levels of the CD4+ TNFR2+ TIL subset tended to correlate with increased tumor growth, with a clear dissociation between the chemotherapy-treated and vehicle-treated tumor-bearing mice (Figure 4(A2)). Then, analyses of the Tregs were performed: the data of Figure 4(B1) indicate that the overall population of CD4+ FOXP3+ Tregs was not affected significantly by chemotherapy. In contrast, the CD4+ TNFR2+ FOXP3+ TILs were significantly reduced by chemotherapy (Figure 4(B1)), indicating that this potent Treg subset was down-regulated by the treatment. Of note, high proportions of both subsets—namely, CD4+ FOXP3+ TILs and CD4+ TNFR2+ FOXP3+ TILs—tended to correlate with elevated tumor volumes that characterized mice that were not treated using chemotherapy (Figure 4(B2,B3)). Furthermore, it was interesting to note that the population of CD4+ TNFR2+ PD-1+ TILs was reduced by chemotherapy (Figure 4(C1)), and tended to correlate with the high tumor volumes that characterized the vehicle-treated tumor-bearing mice (Figure 4(C2)).

**Figure 5:**
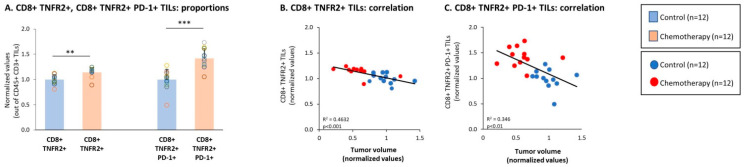
Chemotherapy elevated the proportions of CD8+ TNFR2+ and CD8+ TNFR2+ PD-1+ TILs. Murine 4T1 TNBC cells were syngrafted to BALB/c mice in the presence of chemotherapy or a vehicle control; procedures and analyses were performed as described in Figure 3. (**A**–**C**) CD8+ TNFR2+ and CD8+ TNFR2+ PD-1 TILs, gated out of CD45+ CD3+ cells. (**A**) Proportions of CD8+ TNFR2+ and CD8+ TNFR2+ PD-1+ TILs. (**B**,**C**) CD8+ TNFR2+ and CD8+ TNFR2+ PD-1+ TILs, respectively, correlated with tumor volumes. In the proportion and correlation analyses, the average value of the vehicle-treated group in each biological repeat was given the value of 1 and the values of all mice analyzed in that repeat were normalized accordingly. The value of each mouse is presented as a dot. *** *p* < 0.001, ** *p* < 0.01 (two-tailed unpaired Student’s *t*-test; correlation analyses: Pearson; T.DIST).

In parallel to the reduction in specific CD4+ TNFR2+ TIL subsets, we noted that the CD8+ TNFR2+ subset of TILs was increased significantly by chemotherapy (Figure 5A). Moreover, high proportions of this subset—which were not identified previously in the TNBC mouse tumors—were significantly correlated with smaller tumor volumes that were typical of the chemotherapy-treated mice (Figure 5B). An even more pronounced and significant elevation was induced by chemotherapy in the subset of CD8+ TNFR2+ PD-1+ TILs (Figure 5A), which also demonstrated a significant correlation with the lower tumor sizes of chemotherapy-treated mice (Figure 5C). These findings suggest that protective CD8+ TNFR2+ TILs and CD8+ TNFR2+ PD-1+ TILs became more abundant in the tumors following chemotherapy and that PD-1 expression by these cells may signify that the cells were at the peak of their activation or activity.

**Figure 6:**
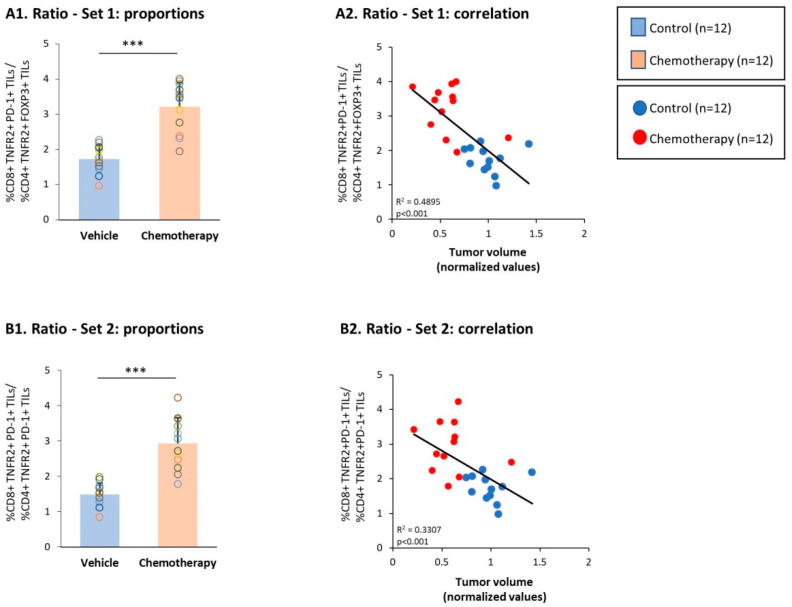
Chemotherapy increased the proportion of CD3+ TNFR2+ PD-1+ TILs at the expense of CD4+ TNFR2+ FOXP3+ and CD4+TNFR2+ PD-1+ TILs. Murine 4T1 TNBC cells were syngrafted to BALB/c mice in the presence of chemotherapy or a vehicle control; procedures and analyses were performed as described in Figure 3. (**A**) Ratio—set 1: ratio between CD8+ TNFR2+ PD-1+ TILs and CD4+ TNFR2+ FOXP3+ TILs. (**A1**) Ratio of CD8+ TNFR2+ PD-1+ TILs over CD4+ TNFR2+ FOXP3+ TILs, calculated on the basis of the percentages of the two subsets in each mouse. (**A2**) The ratio of CD8+ TNFR2+ PD-1+ TILs over CD4+ TNFR2+ FOXP3+ TILs, correlated with tumor volumes. (**B**) Ratio—set 2: ratio between CD8+ TNFR2+ PD-1+ TILs and CD4+ TNFR2+ PD-1+ TILs. (**A1**) Ratio of CD8+ TNFR2+ PD-1+ TILs over CD4+ TNFR2+ PD-1+ TILs, calculated on the basis of the percentages of the two subsets in each mouse. (**A2**) The ratio of CD8+ TNFR2+ PD-1+ TILs over CD4+ TNFR2+ PD-1+ TILs, correlated with tumor volumes. In the proportion and correlation analyses, the average value of the vehicle-treated group in each biological repeat was given the value of 1 and the values of all mice analyzed in that repeat were normalized accordingly. The value of each mouse is presented as a dot. *** *p* < 0.001 (two-tailed unpaired Student’s *t*-test; correlation analyses: Pearson; T.DIST).

The above findings indicated that whereas the CD4+ TNFR2+ FOXP3+ and the CD4+ TNFR2+ PD-1+ TIL subsets were connected to high tumor growth in the absence of chemotherapy treatment, the CD8+ TNFR2+ and particularly the CD8+ TNFR2+ PD-1+ TIL subsets were linked to the inhibition of tumorigenicity in 4T1 tumors when the mice were treated using chemotherapy. Moreover, when two sets of ratio analyses were performed, we found evidence of the importance of the shift between the CD8+ TNFR2+ and CD4+ TNFR2+ subsets. The findings of set 1 (Figure 6A) indicate that the ratio of CD8+ TNFR2+ PD-1+ TILs over CD4+ TNFR2+ FOXP3+ TILs was significantly higher in the chemotherapy-treated mice compared to the vehicle-treated mice, and was significantly associated with reduced tumor volumes. In a similar manner, the findings of set 2 (Figure 6B) indicate that the ratio of CD8+ TNFR2+ PD-1+ TILs over CD4+ TNFR2+ PD-1+ TILs was also significantly elevated following chemotherapy and was significantly connected to restrained tumor growth.

Together, the observations made in this part of the study identified two new TIL subsets—CD8+ TNFR2+ TILs and CD8+ TNFR2+ PD-1+ TILs—in TNBC tumors and shed light on their regulation by chemotherapy. The data indicate that a chemotherapy-induced up-regulation of CD8+ TNFR2+ and CD8+ TNFR2+ PD-1+ TILs at the expense of CD4+ TNFR2+ FOXP3+ TILs and CD4+ TNFR2+ PD-1+ TILs was connected to reduced tumor growth, possibly through alteration in anti-tumor immune activities.

### 3.4. Chemotherapy Led to Down-Regulation of CD4+ TNFR2+ Subsets in Spleens of TNBC-Bearing Mice, but Did Not Give Rise to Up-Regulation of CD8+ TNFR2+ Subsets in Their Spleens

**Figure 7:**
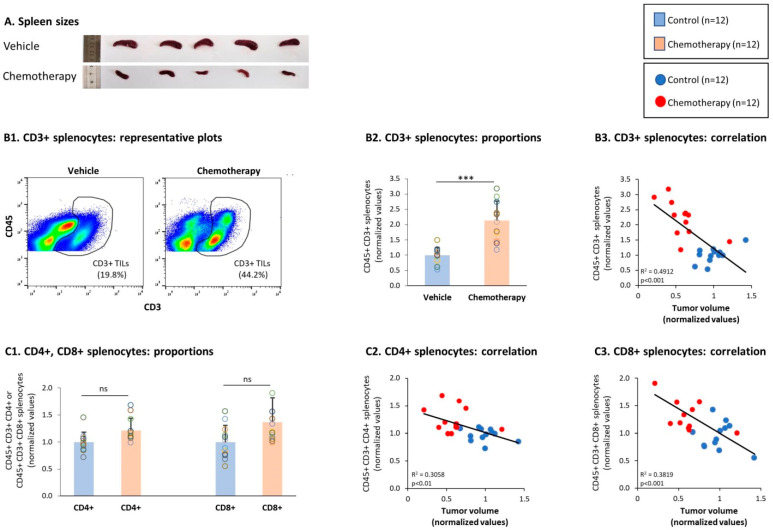
Chemotherapy elevated the proportions of CD3+ splenocytes but did not have a significant impact on the proportions of CD4+ and CD8+ splenocytes. Murine 4T1 TNBC cells were syngrafted to BALB/c mice in the presence of chemotherapy or a vehicle control; procedures and analyses were performed as described in Figure 3. (**A**) Spleen sizes. Spleens excised at the end of the third experiment are demonstrated: in the two groups, there were n = 5 mice in each group. (**B**) CD3+ splenocytes, gated out of CD45+ cells. (**B1**) Representative flow cytometry analyses of CD3+ splenocytes. (**B2**) Proportions of CD3+ splenocytes. (**B3**) CD3+ splenocytes correlated with tumor volumes. (**C**) CD4+ splenocytes and CD8+ splenocytes, gated out of CD45+ CD3+ cells. (**C1**) Proportions of CD4+ and CD8+ splenocytes. (**C2**,**C3**) CD4+ splenocytes and CD8+ splenocytes, respectively, correlated with tumor volumes. In the proportion and correlation analyses, the average value of the vehicle-treated group in each biological repeat was given the value of 1 and the values of all mice analyzed in that repeat were normalized accordingly. The value of each mouse is presented as a dot. *** *p* < 0.001, ns: not significant (two-tailed unpaired Student’s *t*-test; correlation analyses: Pearson; T.DIST).

Previous studies have demonstrated that in many cancer systems, the development of tumors—including 4T1 TNBC tumors—is accompanied by splenomegaly due to increased infiltration of myeloid cells induced by tumor-derived secreted factors [62]. Thus, it is expected that in parallel to reducing tumor volumes, chemotherapy would also lead to a substantial reduction in spleen sizes. Indeed, the data presented in Figure 7A demonstrate that alongside its ability to down-regulate tumor volumes (Figure 2A), chemotherapy led to a prominent reduction in spleen sizes (Figure 7A).

The chemotherapy-induced reduction in spleen sizes was accompanied by elevated levels of CD3+ T cells in the spleens of 4T1-bearing mice (Figure 7(B1,B2)). Moreover, the elevated levels of CD3+ T cells in spleens of chemotherapy-treated mice were significantly correlated with their reduced tumor volumes (Figure 7(B3)). In parallel, we found that the proportions of CD4+ T cells and CD8+ T cells in the spleens were elevated by chemotherapy though not in a significant manner (Figure 7(B1)); yet, the increased levels of these two subsets following chemotherapy were significantly correlated with reduced tumor growth (Figure 7(C2,C3)), suggesting that chemotherapy affected CD4+ and CD8+ T cells in the direction of anti-tumor phenotypes.

**Figure 8:**
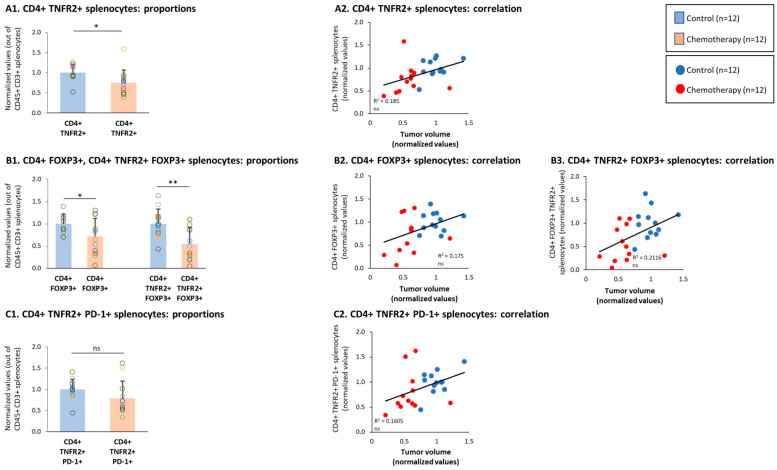
Chemotherapy reduced the proportions of CD4+ TNFR2+ FOXP3+ and CD4+ TNFR2+ PD-1+ splenocytes. Murine 4T1 TNBC cells were syngrafted to BALB/c mice in the presence of chemotherapy or a vehicle control; procedures and analyses were performed as described in Figure 3. (**A**) CD4+ TNFR2+ splenocytes, gated out of CD45+ CD3+ cells. (**A1**) Proportions of CD4+ TNFR2+ splenocytes. (**A2**) CD4+ TNFR2+ splenocytes, correlated with tumor volumes. (**B**) CD4+ FOXP3+ and CD4+ TNFR2+ FOXP3+ splenocytes, gated out of the CD45+ CD3+ cells. (**B1**) Proportions of CD4+ FOXP3+ and CD4+ TNFR2+ FOXP3+ splenocytes. (**B2**,**B3**) CD4+ FOXP3+ and CD4+ TNFR2+ FOXP3+ splenocytes, respectively, correlated with tumor volumes. (**C**) CD4+ TNFR2+ PD-1+ splenocytes, gated out of the CD45+ CD3+ cells. (**C1**) Proportions of CD4+ TNFR2+ PD-1+ splenocytes. (**C2**) CD4+ TNFR2+ PD-1+ splenocytes, correlated with tumor volumes. In the proportion and correlation analyses, the average value of the vehicle-treated group in each biological repeat was given the value of 1 and the values of all mice analyzed in that repeat were normalized accordingly. The value of each mouse is presented as a dot. ** *p* < 0.01,* *p* < 0.05, ns: not significant (two-tailed unpaired Student’s *t*-test; correlation analyses: Pearson; T.DIST).

Then, additional analyses demonstrated that the spleen subsets of CD4+ TNFR2+, CD4+ FOXP3+, and CD4+ TNFR2+ FOXP3+ T cells were all significantly down-regulated upon chemotherapy treatments (Figure 8(A1,B1)) and tended to correlate with higher tumor volumes (Figure 8(A2,B2,B3)); the subset of CD4+ TNFR2+ PD-1+ splenocytes showed a tendency toward down-regulation following chemotherapy, though not in a significant manner (Figure 8(C1,C2)). Thus, in general, these CD4+ subsets demonstrated the same chemotherapy-induced regulatory pattern that was revealed in the TILs.

**Figure 9:**
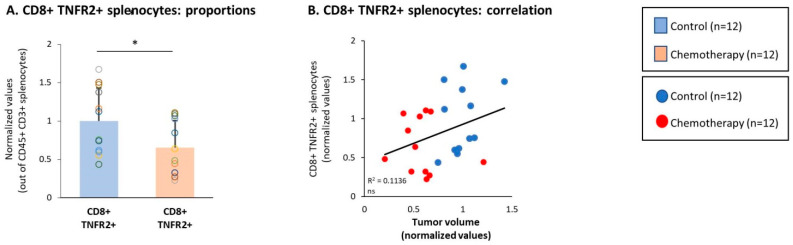
Chemotherapy reduced the proportion of CD8+ TNFR2+ splenocytes, accompanied by the absence of CD8+ TNFR2+ PD-1+ splenocytes. Murine 4T1 TNBC cells were syngrafted to BALB/c mice in the presence of chemotherapy or a vehicle control; procedures and analyses were performed as described in Figure 3. CD8+ TNFR2+ splenocytes, gated out of the CD45+ CD3+ splenocytes. (**A**) Proportions of the CD8+ TNFR2+ splenocytes. (**B**) CD8+ TNFR2+ splenocytes correlated with tumor volumes. CD8+ TNFR2+ PD-1+ splenocytes were not detected prior to or after chemotherapy (data not shown). In the proportion and correlation analyses, the average value of the vehicle-treated group in each biological repeat was given the value of 1 and the values of all mice analyzed in that repeat were normalized accordingly. The value of each mouse is presented as a dot. * *p* < 0.05, ns: not significant (two-tailed unpaired Student’s *t*-test; correlation analyses: Pearson; T.DIST).

A different observation was then made with respect to the effects of chemotherapy on CD8+ TNFR2+ splenocytes. Here, in marked contrast to the chemotherapy-induced increase in CD8+ TNFR2+ TILs in tumors (Figure 5A), CD8+ TNFR2+ splenocytes were reduced by chemotherapy (Figure 9A) and their high levels tended to correlate with increased tumor volumes (Figure 9B). Of major interest was the fact that the CD8+ TNFR2+ PD-1+ subset—which was not detected in the spleens prior to chemotherapy (Figure 2B2)—was also not present after chemotherapy (data not shown), thus it was not increased by the treatment, in contrast to the findings for the TILs (Figure 5A).

Overall, the findings of this part of the study revealed that, in general, chemotherapy induced similar effects on CD3+ and CD4+ TNFR2+ T cells in TILs and splenocytes; however, major differences were revealed between the chemotherapy-driven effects on CD8+ T cells in TILs and splenocytes: the CD8+ TNFR2+ and CD8+ TNFR2+ PD-1+ subsets, which were increased by chemotherapy in the tumors, were either reduced (CD8+ TNFR2+) or not present at all (CD8+ TNFR2+ PD-1+) following chemotherapy treatment in the spleens.

## 4. Discussion

It has long been established that the tumor microenvironment exerts important roles in regulating tumor progression in a very large variety of malignancies. Within the tumor milieu, the potent inflammatory cytokine TNFα has been the subject of intensive investigation. Although anti-tumor functions were described for TNFα when it was administered locally at high doses to patients and cancer-bearing animals, the chronic presence of TNFα in tumors during their progression was strongly connected to a more aggressive disease phenotype in many tumor types [39,42,63,64,65,66,67]. In breast cancer, for example, TNFα was found to be expressed in patient tumors from early stages of tumor development and its elevated levels were associated with more advanced disease and with recurrence [68,69,70]. TNFα was also found to exert a large variety of metastasis-promoting activities, on breast cancer cells themselves and their surroundings [39,65,66,67].

The well-identified tumor-promoting roles of TNFα suggested that this cytokine is an appropriate target for therapy in cancer. Along the same lines, different studies proposed that novel therapeutic measures in cancer could be directed to the TNFα receptors, TNFR1 and TNFR2. Studies of TNFR2 expression by lymphocytes have supported the potential use of TNFR2 as a target for therapy in cancer by demonstrating that CD4+ TNFR2+ Tregs were connected to elevated disease progression in malignancy [31,32,33,34,35,36,37,38].

However, our recent study on TNFR2 TILs revealed much more complex roles for TNFR2 in TNBC [44]. In contrast to other cancer types, we found that TNFR2+ TILs were significantly associated with improved survival in TNBC patients [44]. The extent of TNFR2+ TILs in these patients was identified in biopsies taken prior to chemotherapy, but survival rates were determined following chemotherapy. Thus, we hypothesized that different subsets of TNFR2+ TILs that were present in the tumors prior to chemotherapy were affected by the treatment, eventually leading to improved immune surveillance and better prognosis of TNBC patients. Such a process could be further enhanced by the ability of chemotherapy to generate or expose neo-antigens (as was reported before [71,72]), which could have marked the tumor cells for attack by CD8+ T cells.

To follow up on this hypothesis, we set the current study in which we identified the impact of chemotherapy on the phenotype of TNFR2-expressing TILs of the CD4 and CD8 arms. After validating that indeed chemotherapy was effective at reducing the tumor mass, we found that the proportions of CD3+ TILs and of CD8+ TILs—which were expected to be cytotoxic T cells—were elevated by chemotherapy; these findings supported the possibility that chemotherapy induced immune surveillance, leading to reduced tumor cell growth.

Then, when we analyzed different TNFR2+ TIL subsets, where our observations indicated that chemotherapy had up-regulated the proportions of previously unidentified TIL sub-populations in TNBC consisting of CD8+ TNFR2+ and CD8+ TNFR2+ PD-1+ TILs. Moreover, after chemotherapy, elevated levels of these two TIL subsets were significantly correlated with lower tumor volumes. These novel findings suggest that CD8+ TNFR2+ and CD8+ TNFR2+ PD-1+ TILs were beneficial T cells that contributed to anti-tumor immunity and reduced tumor growth in TNBC. This possibility is strongly supported by a recent report analyzing TILs in TNBC patients, demonstrating that a high density of CD8+ PD-1+ TILs was significantly associated with improved disease-free survival in the patients [54]. Studies in other tumor systems also pointed at the presence of immune-reactive CD8+ PD-1+ T cells in the tumors [60,61].

In view of the above findings [54,60,61] and the elusive nature of PD-1—which can identify T cells that are at their highest level of activation under certain conditions—it is possible that the CD8+ TNFR2+ PD-1+ TILs that we identified in the chemotherapy-treated TNBC tumors consisted of activated/effector cytotoxic T cells that could exert anti-tumor activities and lead to reduced tumor growth. This option gains support given that this unique subset was elevated after chemotherapy in the tumors, but was absent in spleens of chemotherapy-treated mice; these findings raise the possibility that CD8+ TNFR2+ PD-1+ lymphocytes were generated in the spleens but were recruited to tumors by chemotactic factors, and that this process was enhanced by chemotherapy.

In parallel to its ability to up-regulate the CD8+ TNFR2+ and CD8+ TNFR2+ PD-1+ subsets, chemotherapy reduced the levels of two CD4+ TNFR2+ subsets: CD4+ TNFR2+ FOXP3+ and CD4+ TNFR2+ PD-1+ TILs. Moreover, the lower presence of these two CD4+ TNFR2+ sub-populations after chemotherapy tended to associate with reduced tumor growth. These findings suggest that CD4+ TNFR2+ TILs had tumor-promoting activities and that their reduction via chemotherapy contributed to lower tumor growth. This possibility is supported by the higher presence of CD4+ TNFR2+ FOXP3+ and CD4+ TNFR2+ PD-1+ cells in the tumors of vehicle-treated mice compared to their spleens, suggesting that in the lack of chemotherapy, these two cell types were recruited into the tumors and exerted tumor-promoting activities.

The CD4+ TNFR2+ FOXP3+ subset was previously characterized as a population of potent Tregs [18,19,20,21,22,23]; therefore, our findings emphasize the important effect of chemotherapy, which reduced the presence of detrimental immuno-suppressive lymphocytes in the tumors. The identity of CD4+ TNFR2+ PD-1+ cells that were increased by chemotherapy is elusive at this stage. In view of the importance of Th1/Th2 balance in breast cancer progression [73,74], our findings suggest that CD4+ TNFR2+ PD-1+ were either exhausted Th1 cells or activated Th2 cells, whose levels were reduced by chemotherapy.

We found it particularly important that the ratio of CD8+ TNFR2+ PD-1+ TILs over either of these two subsets of CD4+ TNFR2+ TILs (CD4+ TNFR2+ FOXP3+ and CD4+ TNFR2+ PD-1+) was significantly increased in chemotherapy-treated mice and was strongly connected to reduced tumor volumes. These findings suggest that the chemotherapy-driven dominance of CD8+ TNFR2+ TILs at the expense of deleterious CD4+ TNFR2+ TILs had a beneficial impact on tumor growth in TNBC tumors. The high proportions of CD8+ TILs—denoted in the TNBC patient biopsies prior to chemotherapy (Figure 1) and in 4T1 tumors not treated by chemotherapy (Figure 2)—suggest that chemotherapy selected for existing CD8+ TNFR2+ TILs or promoted their proliferation at the tumor site. A non-mutually-exclusive scenario is that CD8+ TNFR2+ PD-1+ cells that were activated in the secondary lymphoid organs were recruited to the tumors following chemotherapy, as suggested above based on the comparisons between the proportions of CD8+ TNFR2+ PD-1+ TILs and CD8+ TNFR2+ PD-1+ splenocytes.

Eventually, the result was that the balance between CD8+ TNFR2+ TILs and CD4+ TNFR2+ TILs shifted at the TME and could lead to the predominance of beneficial cytotoxic CD8+ TILs. These findings provide evidence for the complex nature of TNFR2+ lymphocytes at the TME and emphasize the need to consider treatments that are directed to TNFR2+ TILs as a therapeutic measure with caution. Moreover, they call for a follow-up determination of the specific nature of different CD8+ TNFR2+ TILs and CD4+ TNFR2+ TIL sub-populations in various types of tumors. Accordingly, in further studies on mouse TNBC systems, we will explore the impact of chemotherapy on the identity and functions of different CD8+ TNFR2+ and CD4+ TNFR2+ TIL subsets, determine whether they are exhausted or activated cells, uncover their site of generation and modes of migration, and investigate the dynamics of these processes.

Independently of such further research, our findings may have important clinical implications in terms of therapy considerations in TNBC. Combined with our published study that connected TNFR2+ TILs with improved survival in TNBC patients, the results of the current TNBC animal model system suggest that modalities that target TNFR2+ TILs may have a harmful effect on TNBC. The treatment of choice given to TNBC patients is chemotherapy, which may lead to the selection of beneficial cytotoxic CD8+ TNFR2+ TILs, while reducing unfavorable subsets of CD4+ TNFR2+ TILs. By administering TNFR2 inhibitors, this delicate equilibrium that was induced by chemotherapy and benefits the patients will be interrupted and may lead to detrimental outcomes and a worse prognosis.

## Figures and Tables

**Table 1:** Clinicopathological characteristics of TNBC patients included in the study (n = 41).

Clinicopathological Characteristics of TNBC Cases (n = 41)		
Median Age at Dx (Range), Years: 53 (30–84)		
	Number (n)	Percent (%)
Tumor stage		
pT1	26	63.4
pT2	14	34.1
pT3	1	2.4
Lymph node involvement		
pN0	28	68.3
pN1	11	26.8
pNx	2	4.9
Tumor grade		
2	5	12.2
3	35	85.4
NA	1	2.4

NA, not available.

## Data Availability

Not applicable.

## Supplementary Materials

The following are available online at https://www.mdpi.com/article/10.3390/cells10061429/s1, Figure S1: Gating strategies in the analysis of TILs and splenocytes, Figure S2: Subset analysis of CD4+ and CD8+ T cells, Figure S3: Membrane PD-L1 expression by 4T1 murine TNBC cells.
