# Supplementary material for: Chemotherapy Shifts the Balance in Favor of CD8+ TNFR2+ TILs in Triple-Negative Breast Tumors
Source: Cells. 2021 Jun 8;10(6):1429. doi: 10.3390/cells10061429 (PMC8229590; doi:10.3390/cells10061429)
Supplement: Supplementary file 1 [file cells-10-01429-s001.zip › cells-1220576-supplementary.pdf]

Figure S1

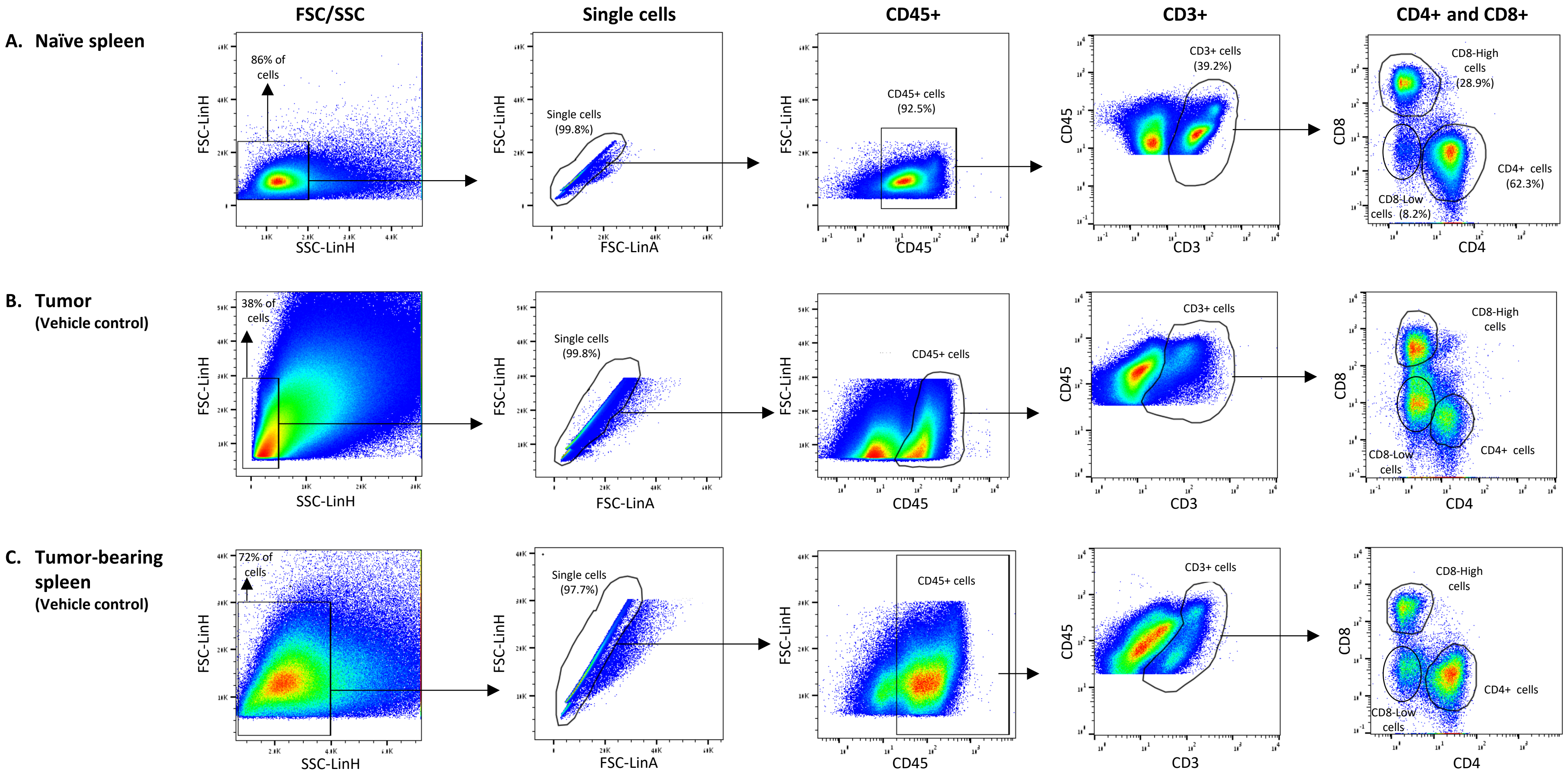

Gating strategies in the analysis of TILs and splenocytes

The Figure demonstrates the gating strategy taken in analyses of lymphocyte subsets in the study. **Additional information on procedures of cell acquisition and gating is provided in "Materials and Methods".** Naive mouse spleens, mouse tumors and tumor-bearing mouse spleens were dissociated; following staining, fixed cells were analyzed for the expression of different markers. **(A)** A sample of spleen of naïve 9-weeks old mouse (not injected with tumor cells), demonstrating the validity of the gating strategy. **(B)** Analysis of cells isolated from a tumor of a vehicle-treated mouse. Because the data are from one representative mouse, the percentages of CD45+, CD3+, CD4+ and CD8+ subsets are not indicated in the three right columns of Part (B). The same procedure was taken for all mice in the study. The CD45 panel contains a relatively large proportion of CD45-negative cells, which are presumably mainly tumor cells. **(C)** Analysis of cells isolated from a spleen of a vehicle-treated tumor-bearing mouse. Because the data are from one representative mouse, the percentages of CD45+, CD3+, CD4+ and CD8+ subsets are not indicated in the three right columns of Part (C). The same procedure was taken for all mice in the study. In spleens of naïve mice (Part A), a small population with low CD8 expression was identified; This subset was noted also in tumors and spleens of tumor-bearing mice, treated by chemotherapy or by vehicle; because this subset of CD8-Low cells occasionally overlapped with the unstained population in control staining, further analyses were performed only on the CD8-High sub-population.

Figure S2

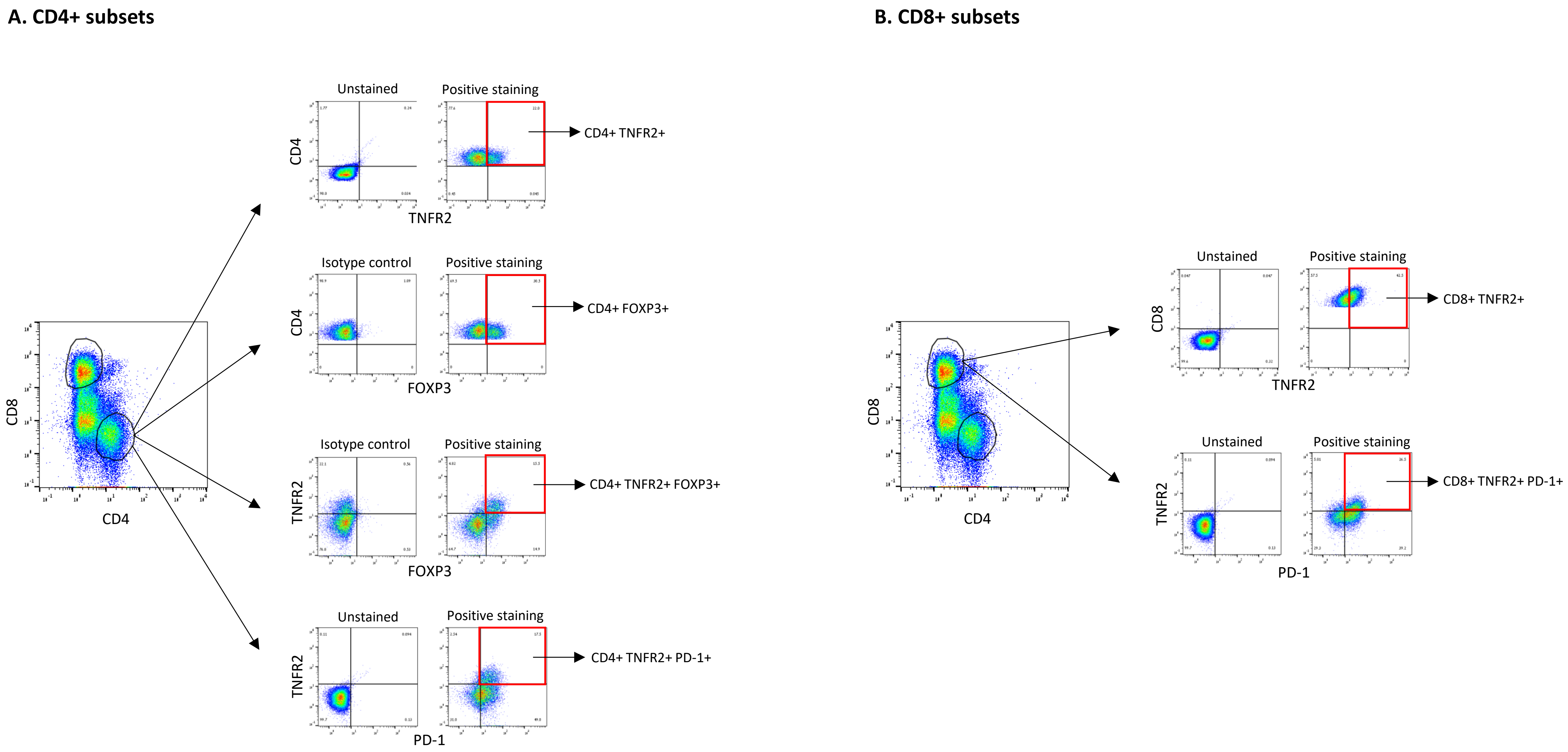

Subset analysis of CD4+ and CD8+ T cells

The Figure demonstrates the gating strategy of CD4+ and CD8+ subsets in fixed cells, exemplified on TILs from a representative vehicle-treated 4T1-injected mouse; a similar strategy was taken for all mice included in the study. **(A)** CD4+ cells. **(B)** CD8+ cells. The proportions of the following sub-populations were determined (in red squares): (A) CD4+ TNFR2+, CD4+ FOXP3+, CD4+ TNFR2+ FOXP3+, CD4+ TNFR2+ PD-1+. (B) CD8+ TNFR2+ and CD8+ TNFR2+ PD-1+. Marker-positive cells were determined using unstained cells tuned to the same fluorophore channels, or cells stained with non-relevant isotype control in the case of FOXP3.

Figure S3

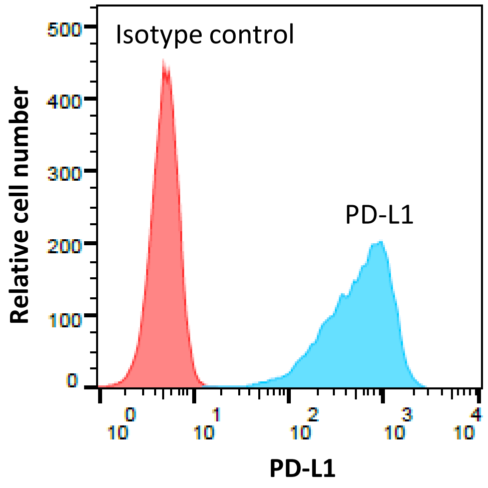

Membrane PD-L1 expression by 4T1 murine TNBC cells
Murine 4T1 TNBC cells were infected to express mouse PD-L1, determined by flow cytometry. A representative experiment of n=2 is demonstrated.
